# Defective defence in *Daphnia* daughters: silver nanoparticles inhibit anti-predator defence in offspring but not in maternal *Daphnia magna*

**DOI:** 10.1038/s41598-020-64652-7

**Published:** 2020-05-15

**Authors:** Sarah Hartmann, Anna Beasley, Darya Mozhayeva, Carsten Engelhard, Klaudia Witte

**Affiliations:** 1 Research Group of Ecology and Behavioural Biology, Institute of Biology, Department of Chemistry-Biology, University of Siegen, Adolf-Reichwein-Strasse 2, Siegen, 57076 Germany; 2 Faculty of Biology, Medicine and Health, University of Manchester, Oxford Road, Manchester, M13 9PL United Kingdom; 3 Department of Chemistry-Biology, University of Siegen, Adolf-Reichwein-Strasse 2, Siegen, 57076 Germany

**Keywords:** Ecology, Zoology, Ecology, Environmental sciences, Limnology

## Abstract

One major environmental problem of our time are emerging contaminants in the aquatic environment. While nanoparticles exhibit attractive features such as antimicrobial properties in the case of silver nanoparticles (AgNPs), earlier studies suggest that NPs are not completely filtered out at wastewater treatment plants and may therefore be continuously introduced into the aquatic environment. Although adverse effects of AgNPs on aquatic organisms have been extensively studied, there is still a lack of knowledge on how this chemical stressor interacts with natural cues on the maternal and subsequent generation of aquatic organisms. We tested whether AgNPs (NM-300K, 14.9 ± 2.4 nm, concentration range: 2.5 µg/L – 20 µg/L) affect the kairomone-induced adaptive anti-predator defence mechanism in maternal *Daphnia* and their offspring. While maternal *Daphnia* developed typical anti-predator defence mechanisms when exposed to kairomones and AgNPs, their offspring could not develop such adaptive defensive traits. The lack of this defence mechanism in offspring could have dramatic negative consequences (e.g. reduced *Daphnia* population) for the entire complex food web in the aquatic ecosystem. For a realistic risk assessment, it is extremely important to test combinations of chemical stressors because aquatic organisms are exposed to several natural and artificial chemical stressors at the same time.

## Introduction

Since the end of the 18th century, the industrial revolution has led to enormous technical, health and economic improvements for human welfare. However, technological progress is interfering with global cycles that could lead to negative changes in the environment^1^. One major environmental problem of our time is the environmental pollution caused by mankind^1^. In recent decades, pollution of the aquatic environment has risen to new levels^2^ due to the release of synthetic or natural-occurring compounds found in pharmaceuticals, personal care products, industrial and household products, metals, and nanomaterials into aquatic ecosystems^1,2^. One of the most commonly used nanomaterials are silver nanoparticles (AgNPs) due to their antimicrobial properties. Many medical products, such as wound dressings, bandages and sanitation devices use AgNPs^3^. In addition, common household objects, food containers, and sports clothing contain AgNPs, and even washing machines are impregnated with AgNPs to reduce bacterial growth and odour^3^. Based on their small size (less than 100 nm in size in one dimension), NPs are not completely filtered out at waste-water treatment plants (approximately 50 to 99% removal efficiency with regional variations and depending on the type of NP)^4^, and a significant amount of Ag-containing NPs is still continuously released into freshwater ecosystems^5^. Maurer-Jones *et al*.^6^ estimated that the predicted environmental concentrations (PECs) for AgNPs in surface water range from 0.088 to 10,000 ng/L.

Besides the numerous studies on the negative effects of high concentrations of AgNPs on aquatic organisms such as *Daphnia*^7–11^, it was shown that AgNPs affect aquatic organisms even at low, environmentally relevant concentrations. Hartmann *et al*.^12^ reported that low concentrations of AgNPs (1.25–10 µg/L) lead to a significant reduction in reproductive success in *Daphnia magna*, a key species within the complex aquatic food web and a standard model species for ecotoxicological studies^13–15^. Chronic exposure of *Daphnia similis* to PVP-coated AgNPs (0.02 and 1 µg/L) inhibited reproduction due to a down regulation of key fatty acids which are required for egg production, larval development and environmental sex determination^16^. Zhao and Wang^17^ reported a significant reduction in body length in adult *D. magna* when exposed to AgNPs (carbonate-coated) at a concentration of less than 5 μg/L.

Although many effects of AgNPs on aquatic organisms are well studied, there is still a lack of knowledge on the interaction of NPs with natural chemical stressors in water systems and how this interaction may affect aquatic organisms. For example, kairomones are chemical stimuli emitted by a predator, which indicates the presence of that predator to the prey. Pokhrel and Dubey^18^ assumed that on one hand the presence of NPs inhibits the predator to release kairomones into the water and on the other hand NPs might result in a lack of a predator induced response by prey as low concentrations of citrate-AgNPs (2 µg/L) defect the sensory system of *Daphnia*^18^. To the best of our knowledge, effects of AgNPs on a kairomone-mediated anti-predator defence in *Daphnia spp*. and their offspring have never been investigated. Whether AgNPs affect the kairomone-induced anti-predator defence in *Daphnia spp*. or not is very important to know because in aquatic systems *Daphnia* are exposed to both chemical stressors simultaneously. Furthermore, investigating the effect of the combined stressors is a much more realistic scenario and will lead to a better risk assessment of AgNPs in the environment. *Daphnia* is an excellent model species to investigate the development of defensive traits in response to the presence of predators indicated by kairomones^19^ and to the presence of AgNPs. It has been shown several times that in the presence of a predator species, many species of *Daphnia* change life history, behavioural and morphological traits^20,21^. The kairomone-mediated response in *Daphnia* includes growth of defensive morphological traits, e.g. growth of a helmet^22^, neckteeth^23,24^, a crown of thorns^25^, an elongated tail spine and an increase in overall body size^21^. Typical predators for *Daphnia* are the phantom midge larvae *Chaoborus*, the heteropteran *Notonecta* sp. or small fishes^26–28^. In the presence of fish predators, *Daphnia* react with an earlier sexual maturity, an increased fecundity and the production of resting eggs^26,29,30^. The presence of predators even leads to new defensive traits in the next generation. These protected neonates have a better chance of survival from the moment they are born^31^. Offspring of adult *Daphnia* exposed to predatory fish kairomones develop a longer tail spine relative to their total body length than offspring of adult *Daphnia* that were not exposed to fish kairomones^27^.

Thus, the aim of this study was to test whether maternal *Daphnia* exposed to kairomones released from a fish predator and exposed to different environmentally relevant concentrations of AgNPs (NM-300K) are able to develop defensive morphological traits, and/or whether the simultaneous exposure of the maternal generation to kairomones and to different concentrations of AgNPs would lead to adaptations in the offspring or not. We tested different low concentrations of AgNPs to cover a spectrum of possible environmentally relevant contaminations and to exclude single concentration effects. If AgNPs inhibit a predator induced defence in maternal *Daphnia* and/or offspring, this would have dramatic impacts on *Daphnia* populations and therefore on the entire complex food web in the aquatic environment with *Daphnia* as a key species in that food web.

## Results

**Figure 1:**
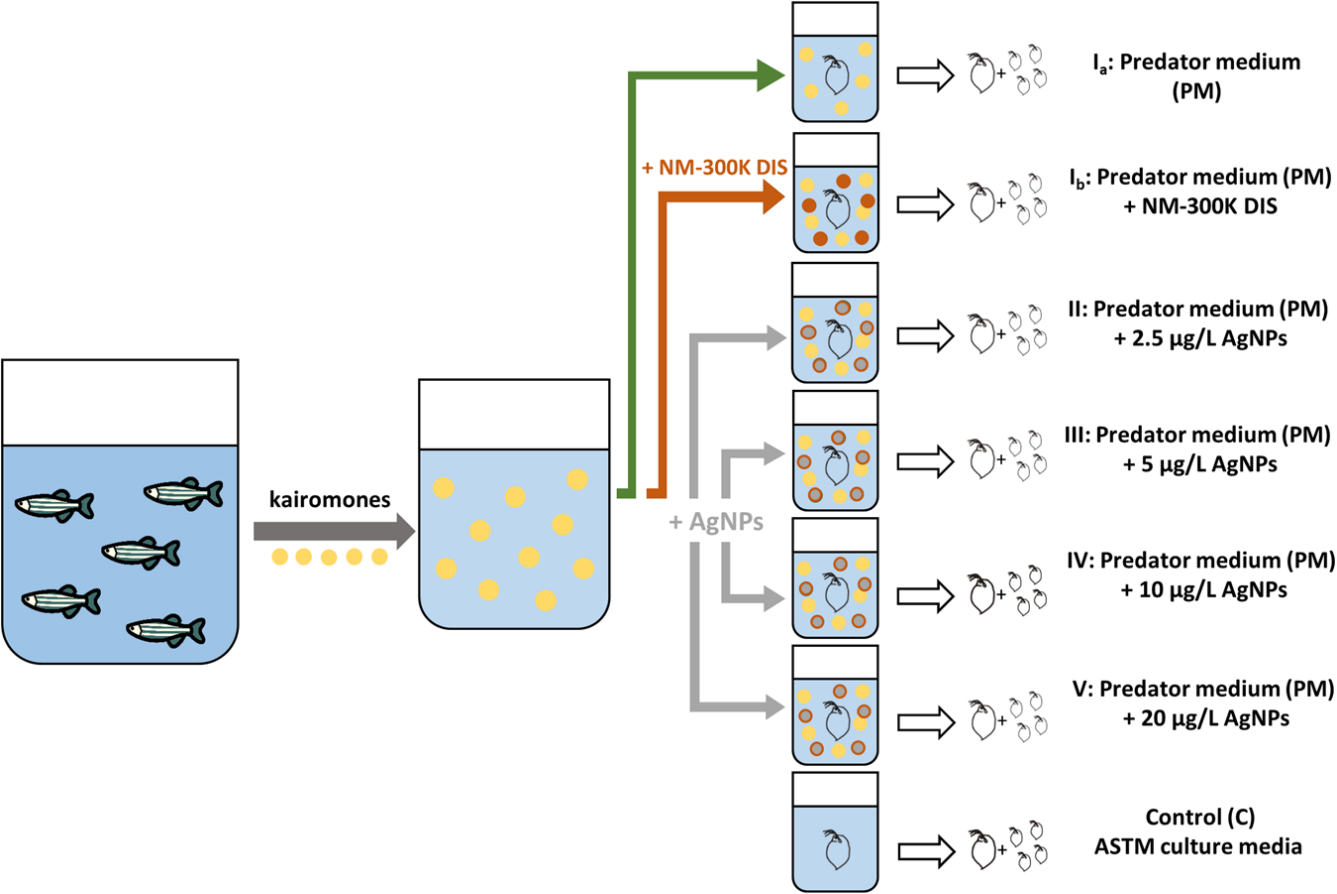
Illustration of the experimental set-up. Treatments were as follows: maternal *D. magna* exposed to predator medium only (PM, Treatment I_a_), exposed to predator medium and NM-300K DIS (Treatment I_b_), exposed to kairomones and different concentrations of AgNPs (Treatment II - V), and maternal *D. magna* exposed to ASTM culture medium which served as a control (C). Yellow dots indicate kairomones released from zebrafish (*D. rerio)*, orange dots indicate NM-300K DIS, and grey dots with an orange covering indicate AgNPs stabilized with NM-300K DIS. Within each Treatment, we analysed maternal *D. magna* and their released offspring.

We found that offspring of maternal *D. magna* which had been exposed to kairomones released from zebrafish, *Danio rerio*, and simultaneously exposed to AgNPs of different environmentally relevant concentrations [2.5, 5, 10 and 20 µg/L] (Treatments II-V, Fig. 1), did not develop a typical defence mechanism as compared to offspring of maternal *Daphnia* which had been exposed to kairomones only (Treatment I_a+b_) (Tables 1 and 2). Instead, offspring of maternal *Daphnia* which had been exposed to kairomones and different concentrations of AgNPs had a smaller relative spine length as compared to the other offspring (Tables 1 and 2).

**Table 1:** Mean body length (mm ± sd), mean spine length (mm ± sd) and relative spine length (% ± sd) of maternal *Daphnia magna* (n = 12) at the end of the experiment (Day 21) and their offspring (n > 1000).

Treatment	Offspring			Maternal *Daphnia*		
	mean body length (mm ± sd)	mean spine length (mm ± sd)	mean relative spine length (% ± sd)	mean body length (mm ± sd)	mean spine length (mm ± sd)	mean relative spine length (% ± sd)
Predator medium (PM)	0.91 ± 0.07^#^	0.51 ± 0.04^#^	36.01 ± 1.69^# # #^	4.17 ± 0.12^#^	0.82 ± 0.13^#^	22.47 ± 5.82^# # #^
PM + 2.5 µg/L AgNPs	0.90 ± 0.06	0.51 ± 0.03	35.88 ± 1.69*	4.26 ± 0.07	0.88 ± 0.07	22.24 ± 5.19
PM + 5 µg/L AgNPs	0.92 ± 0.06*	0.52 ± 0.04*	35.72 ± 1.61 ***	4.45 ± 0.29*	0.90 ± 0.10	22.32 ± 5.40
PM + 10 µg/L AgNPs	0.94 ± 0.08*	0.51 ± 0.04	35.83 ± 1.81 **	4.09 ± 0.07	0.82 ± 0.13	22.39 ± 5.20
PM + 20 µg/L AgNPs	0.92 ± 0.08	0.50 ± 0.03*	35.86 ± 1.98*	4.29 ± 0.07	0.93 ± 0.07	22.49 ± 5.05
Control	0.89 ± 0.07	0.49 ± 0.04	35.73 ± 1.90	3.83 ± 0.31	0.66 ± 0.10	20.19 ± 6.73

^#^indicated significant differences between control and predator medium (PM). *showed significant differences between respective treatment and predator medium (PM). ^#^P < 0.05; ^# # #^P < 0.001; *P < 0.05; **P < 0.01; ***P < 0.001.

**Table 2:** GLMM estimates for the effects on relative spine length in offspring and maternal *Daphnia magna*.

**Fixed effects**	**Estimate**	**Standard error**	***t***	**P**
***Offspring - Treatment I - V***				
(Intercept)	3.585	0.001	2402.713	**<0.001**
PM + 2.5 µg/L AgNPs	−0.004	0.001	−2.429	**0.015**
PM + 5 µg/L AgNPs	−0.007	0.001	−3.933	**<0.001**
PM + 10 µg/L AgNPs	−0.005	0.001	−2.705	**0.006**
PM + 20 µg/L AgNPs	−0.007	0.001	−2.202	**0.02**
***Offspring – Treatment C and I***				
(Intercept)	3.574	0.002	1351.248	**<0.001**
Predator medium (PM)	0.009	0.002	3.465	**<0.001**
***Maternal D. – Treatment I - V***				
(Intercept)	3.017	0.082	36.601	**<0.001**
PM + 2.5 µg/L AgNPs	0.000	0.012	0.011	0.991
PM + 5 µg/L AgNPs	0.000	0.012	0.068	0.946
PM + 10 µg/L AgNPs	−0.002	0.012	−0.166	0.868
PM + 20 µg/L AgNPs	0.010	0.012	0.870	0.384
***Maternal D. – Treatment C and I***				
(Intercept)	2.929	0.129	22.602	**<0.001**
Predator medium (PM)	0.115	0.019	5.803	**<0.001**

Significant differences (P < 0.05) between respective treatment and predator medium (PM) and between control and predator medium (PM) are marked in bold. *t* = test s*t*atistics.

We quantified the total Ag content of the test media to verify the nominal Ag concentrations (Table 3). The measured total Ag concentration in the fresh and aged media for PM (Treatment I_a+b_) and Control (Treatment C) were below LOD (<0.1 µg/L). The total Ag concentration of the fresh media samples with PM + AgNPs were 2.2 µg/L (Treatment II), 4.4 µg/L (Treatment III), 9.3 µg/L (Treatment IV), and 18.7 µg/L (Treatment V; Table 3). The total Ag concentration of the aged media for Treatment II, Treatment III, Treatment IV and Treatment V was 2.0 µg/L, 3.1 µg/L, 8.2 µg/L, and 15.5 µg/L, respectively. A S/TEM image of AgNPs (NM-300K) dispersed in ASTM medium, measured directly after the preparation of the stock solution is shown in Fig. S1.

**Table 3:** Concentration of total Ag [µg/L] and expanded uncertainties [U, k = 2] of the respective treatments measured with ICP-MS of freshly prepared media and aged media samples after 24 h of exposure.

**Treatment**	**Nominal** **concentrations** **(µg/L)**	**Mean concentration (µg/L)** ± **U**	
		**Fresh media**	**Aged media**
**I**_**a**_	—	<LOD	< LOD
**I**_**b**_	—	<LOD	< LOD
**II**	2.5	2.2 ± 0.26	2.0 ± 0.2
**III**	5	4.4 ± 0.24	3.1 ± 0.3
**IV**	10	9.3 ± 0.59	8.2 ± 0.5
**V**	20	18.7 ± 0.90	15.5 ± 0.7
**C**	—	<LOD	<LOD

Note: N = 1, n = 10; N = biological replicates; n = internal replicates; LOD = limit of detection.

**Figure 2:**
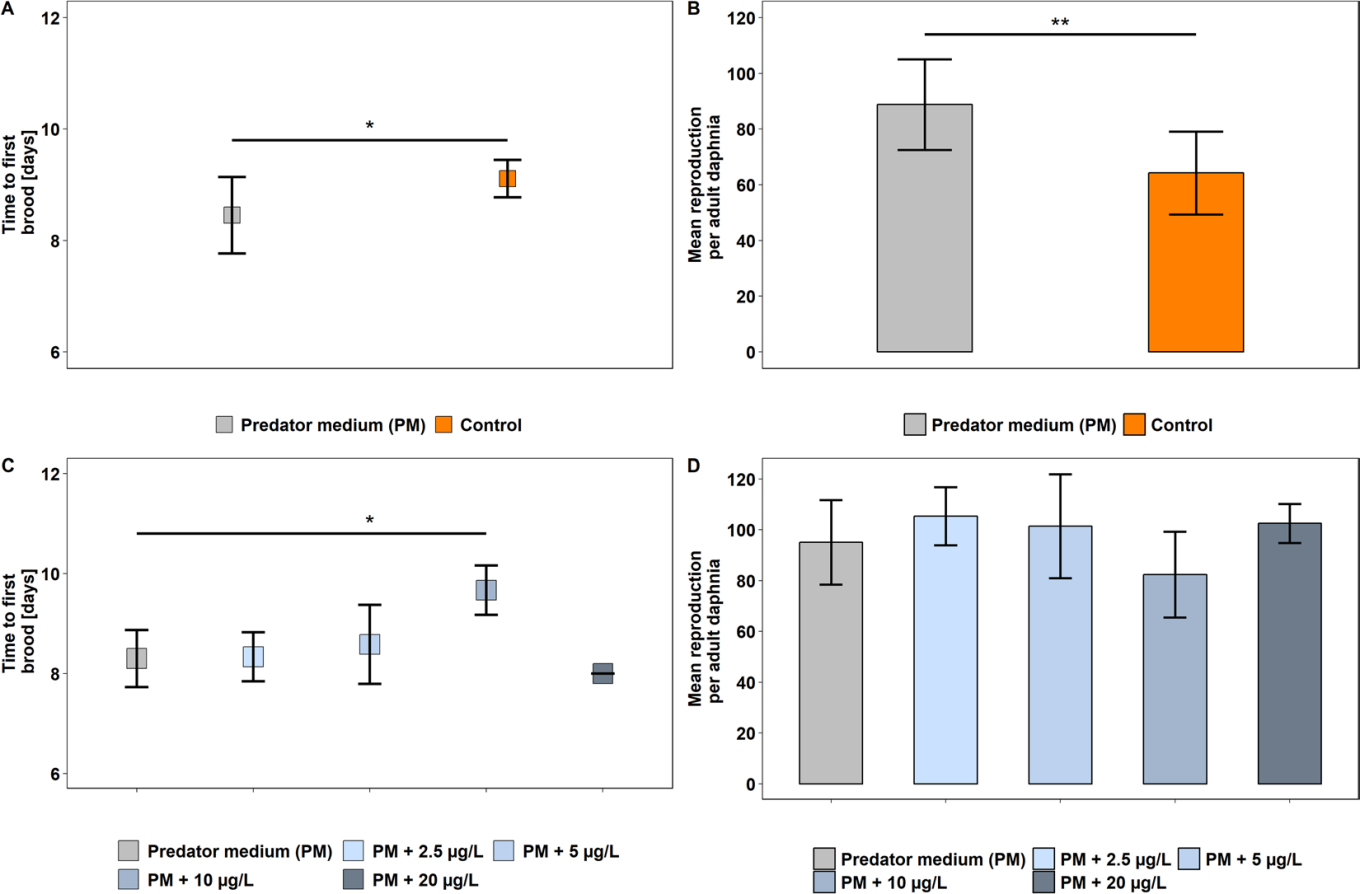
Time to first brood and mean reproduction. Time to first brood [days] ± sd (n = 12 in each treatment) and cumulative mean reproduction ± sd over 21 days of maternal *Daphnia magna* exposed to predator medium (PM) or to ASTM (Control) (**A**,**B**), and maternal *Daphnia* exposed to predator medium (PM) only or to PM + different nominal concentrations of AgNPs (**C**,**D**). *P < 0.05, **P < 0.01.

Maternal *D. magna* exposed to the AgNP-free predator medium (PM; Treatment I_a+b_, Fig. 1) served as a positive control. They changed life history and developed typical defence mechanisms (Fig. 2A + B; Table 1). Maternal *Daphnia* exposed to kairomones (PM) and to different concentrations of AgNPs (Treatment II-V; for more details see Material and Methods section, Fig. 1) simultaneously changed life history and developed defensive traits as well (Fig. 2C + D, Table 1). In the control (C) which served as a general reference, maternal *D. magna* were exposed to the culture medium (ASTM) containing neither kairomones nor AgNPs (Control (C), Fig. 1) and did not change life history pattern and did not develop defensive traits (Fig. 2A + B, Table 1). Because AgNPs were dissolved and stabilized with NM-300K DIS, we exposed maternal *D. magna* to NM-300K DIS and PM to test for any effects from the solvent (Treatment I_b_, Fig. 1). Because we found no differences in any of the measured parameters between maternal *Daphnia* in Treatment I_a_ (PM, Fig. 1) and those in Treatment I_b_ (PM + NM-300K DIS, Fig. 1) we combined these data for further comparison as Treatment I (data not shown).

We measured and analysed the time taken to first brood and reproductive success (as number of offspring), body length (mBL), spine length (mSL) and relative spine length (mRSL) of maternal *Daphnia* in all treatments. Additionally, we measured and analysed the body length (oBL), spine length (oSL), and relative spine length (oRSL) of offspring in all treatments. Maternal *D. magna* exposed to kairomones only (PM, Treatment I, Fig. 1) reproduced significantly earlier (Kruskal-Wallis-test and Dunn’s test, χ² = 6.131, P < 0.01, Fig. 2A), produced a significantly larger number of offspring (one-way ANOVA and Dunnett’s test, P < 0.01, Fig. 2B), developed a significantly larger body length (mBL) (Kruskal-Wallis-test and Dunn’s test, χ² = 7.491, P < 0.01, Table 1), a significantly larger spine length (mSL) (Kruskal-Wallis-test and Dunn’s test, χ² = 6.687, P < 0.01, Table 1) and a significantly larger relative spine length (mRSL) (GLMM, Estimate = 0.115, p < 0.001, Tables 1 and 2) at the end of the experiment (Day 21) in comparison to maternal *Daphnia* in the control (C) with ASTM culture medium only. Similarly, offspring of maternal *Daphnia* in Treatment I, exposed to kairomones only (PM), developed a significantly larger body length (oBL) (Kruskal-Wallis-test and Dunn’s test, χ² = 51.1924, P < 0.01, Table 1), a significantly longer spine length (oSL) (Kruskal-Wallis-test and Dunn’s test, χ² = 122.1717, P < 0.01, Table 1) and a significantly larger relative spine length (oRSL) (GLMM, Estimate: 0.009, p < 0.001, Tables 1 and 2) compared to offspring from maternal *Daphnia* in the control (C). These changes in morphology and in life-history parameters are well described as kairomone-mediated anti-predator defence mechanisms in response to fish predators. Hence, the induction of defensive traits in *D. magna* was successful in maternal *Daphnia* and their offspring, when AgNPs were absent.

Maternal *Daphnia* simultaneously exposed to kairomones and different concentrations of AgNPs did not differ from those exposed to kairomones only (Treatment I) in the time to first brood, with one exception. Maternal *Daphnia* exposed to PM and 10 µg Ag/L reproduced significantly later (mean of 9.7 days) than maternal *Daphnia* exposed to PM only (mean of 8.3 days) (Kruskal-Wallis test with Dunn’s test, χ² = 33.241, P < 0.01, Fig. 2C). The number of offspring did not differ between maternal *Daphnia* exposed to kairomones only and those animals simultaneously exposed to kairomones and different concentrations of AgNPs (Kruskal-Wallis test with Dunn’s test, χ² = 15.928, P > 0.05, Fig. 2D). No concentration dependent pattern was found for maternal *Daphnia* in Treatment I compared to Treatments II-V regarding body length (mBL) and spine length (mSL) after each moult (Table S2).

The most pronounced effects were observed in the offspring of maternal *Daphnia* exposed to kairomones in combination with different environmentally relevant concentrations of AgNPs in Treatments II-V. Offspring in Treatment III (PM and 5 µg Ag/L) and Treatment IV (PM and 10 µg Ag/L) were even larger in body length than offspring in Treatment I (PM) (p < 0.05, Table 1) and thus a more attractive prey to predators. Although offspring of Treatment III and V had longer spines (P < 0.05, Table 1) than offspring in Treatment I, they developed a significantly smaller relative spine length (oRSL) when maternal *D. magna* were exposed to PM and 2.5 µg Ag/L (GLMM, Estimate: − 0.004, p = 0.015, Tables 1 and 2), PM and 5 µg Ag/L (GLMM, Estimate: − 0.007, p < 0.001, Tables 1 and 2), PM and 10 µg Ag/L (GLMM, Estimate: − 0.005 p = 0.006, Tables 1 and 2), and PM and 20 µg Ag/L (GLMM, Estimate: − 0.007, p < 0.05, Tables 1 and 2) compared to offspring born by maternal *Daphnia* exposed to kairomones only (Treatment I, PM). No clear dose response pattern was found for the body length (oBL) and the spine length (oSL) of offspring from maternal *D. magna* exposed to PM and AgNPs in comparison to offspring from Treatment I (PM only) (Table 1).

## Discussion

In this study, we detected a defective kairomone-mediated anti-predator defence mechanism in *Daphnia* daughters when the maternal generation was simultaneously exposed to kairomones from zebrafish *D. rerio* and AgNPs at environmentally relevant low concentrations. Although maternal *D. magna* exposed to kairomones and different concentrations of AgNPs developed typical defensive traits, their offspring did not exhibit such traits. They were in some treatments even larger than those offspring from maternal *Daphnia* exposed to PM only (Treatment I) and they developed a significantly smaller relative spine length which probably makes them even more vulnerable to predators. A smaller relative spine length means that the protection against predators is less efficient for offspring. To the best knowledge of the authors, this is the first study showing that environmentally relevant low concentrations of AgNPs can have a dramatic negative impact on offspring, although they were never directly in contact with these AgNPs (as protected by the brood pouch of maternal *Daphnia*). Our results indicate that maternal *D. magna* are not able to produce offspring with an adaptive defence mechanism against fish predators when exposed to PM and AgNPs. The lack of this effective and adaptive defence mechanism will have a dramatic negative impact on *Daphnia* populations and therefore potentially on the entire food web in the aquatic environment.

In our study, maternal *D. magna* treated with kairomones only (Treatment I, PM) exhibited a typical kairomone-mediated anti-predator defence mechanism as expected. In this Treatment I, the reproductive success of maternal *Daphnia* was significantly higher, they reproduce significantly earlier, and their body length was significantly larger in comparison to maternal *Daphnia* of the control (C) with ASTM-medium only. Thus, our results are in accordance with studies of Barbosa *et al*.^27^ and Ślusarczyk *et al*.^30^ who showed that the exposure to kairomones from fish predators leads to a significant increase in the number of offspring, in body size and to earlier first reproduction of adult *D. magna*. In the presence of fish predators, *Daphnia* invest most of their resources into reproduction than into somatic growth^29^, leading to an early sexual maturity with more but smaller neonates^26^. This predator defence mechanism is adaptive because *D. magna* that sexually mature earlier and with a smaller body size are less conspicuous to fish predators since fish predators select larger prey due to visual hunting^32^. In general, the development of a larger body size from moult to moult is of great benefit for adult *Daphnia*. Due to their larger body size and larger spine helmets or teeth, they are less vulnerable to predators like small fish, e.g. the three-spined stickleback. Due to large defensive traits *Daphnia* which were taken up by the fish were spit out immediately. So far, it is known that environmental pollutants can affect the kairomone-mediated anti-predator defence mechanism in adult *Daphnia*. Trotter *et al*.^19^ found that microplastics inhibit the induction of defensive traits in adult *D. longicephala*, when they were exposed to kairomones of *Notonecta glauca* and plastic waste. Further, Pokhrel and Dubey^18^ showed that adult *Daphnia* treated with low concentrations of citrate-AgNPs and predator medium of the dragonfly *Anax junius*, were not able to detect the presence of the predator in a vertical migration test. The authors assumed that the exposure to AgNPs impairs the sensory system of *Daphnia*, or that the chemoreceptors might be compromised. The chemoreceptors for perception of kairomones are located on the first antennae of *Daphnia*^33^. The chemosensilla of the first antennae are developed already in the juvenile stages of a *Daphnia’s* life cycle, allowing them to detect predators via the chemical signals released into the aquatic environment^22,33^.

In our study, maternal *Daphnia* exposed to kairomones and different low concentrations of AgNPs developed similar defensive traits as maternal *Daphnia* exposed to kairomones only. This is interesting because our previous long-term multi-generation study on *Daphnia* exposed to similar low and environmentally relevant concentrations of AgNPs showed that *Daphnia* reproduced less offspring over six successive generations in comparison to *Daphnia* not exposed to AgNPs^12^. Thus, the presence of AgNPs leads to a reduction in the reproductive success. In the present study, however, the presence of kairomones only led to an increase in the number of offspring, which is the opposite effect. These differences could be explained by a potential change of the AgNPs induced toxicity due to a high content of dissolved organic matter (DOM)^8^. The predator fish swam for about 24 hours within the PM used in this study, which should lead to a significant increase of the DOM content in comparison to the ASTM medium alone. Because we did not measure the DOM in this study, we cannot test this hypothesis. However, it has been reviewed by Zhang *et al*.^34^ that DOM can stabilize AgNPs in aqueous media by blocking oxidative sites, adhering on the surface of AgNPs, and reduce the toxicity of AgNPs to aquatic organisms. Therefore, three main pathways were identified in this review concerning the reduced toxicity of AgNPs in the presence of DOM: protecting organisms from the NPs itself, scavenging free radicals and combining DOM particles with released ionic silver. These findings support the results regarding the reproduction success in the current study. The fact that maternal *Daphnia* exposed to both kairomones and AgNPs reproduced a similarly high number of offspring as maternal *Daphnia* exposed to kairomones only might indicate that the effect of kairomones even prevails the effect of AgNPs.

The fact that maternal *Daphnia* exposed to kairomones and different low concentrations of AgNPs developed similar defensive traits as maternal *Daphnia* exposed to kairomones only, gives a first indication that AgNPs in combination with kairomones had no negative impact on the reproductive success of maternal *Daphnia*. However, we detected a lack of the adaptive kairomone-mediated anti-predator defence mechanism in the offspring of maternal *Daphnia* exposed to both chemical stressors. Even worse, these offspring had a smaller relative spine length than offspring of Treatment I (PM). But why did these offspring not develop typical kairomone-mediated defence mechanisms? A study by Hales *et al*.^35^ found that different gene expression programs are involved in kairomone-mediated anti-predator defence mechanisms in the maternal generation and in offspring of *Daphnia ambigua*, when exposed to kairomones of redbreast sunfish *Lepomis auritus*. The authors provided evidence that the gene expression program within a generation (from moult to moult) and the gene expression program involved in transgenerational responses (from mother to offspring) are distinct programs and regulated independently^35^. Thus, such differences in these two types of gene expression programs might explain, why maternal *Daphnia* responded to kairomones in the presence of AgNPs but their offspring did not. Further studies are required to identify the mechanisms behind this impairment and the role of NPs in gene expression programs in *Daphnia* and other aquatic organisms.

## Conclusion

This study demonstrates for the first time that environmentally relevant, low concentrations of AgNPs in aquatic environments have a negative impact on the adaptive kairomone-mediated anti-predator defence mechanism in *D. magna*. Although maternal *Daphnia* developed typical anti-predator defence mechanisms when exposed to kairomones and AgNPs, their offspring could not develop such adaptive defensive traits. This lack of defence mechanism in offspring of *Daphnia* could therefore have dramatic impacts and consequences on *Daphnia* population structure in the presence of predation risk, and thus on the entire complex food web. Hence, this study provides evidence that an anthropogenic pollutant released into the aquatic environment interfere with evolutionary adaptation strategies in cladoceran. Our study is the first one investigating the effect of two chemical stressors on an evolved anti-predator defence strategy in *Daphnia* with dramatic effects in the offspring. This shows that it is extremely important to test a combination of chemical stressors simultaneously instead of testing them separately. Our approach is a more realistic exposure scenario for an aquatic organism which would typically be exposed to several natural and man-made chemical stressors at the same time. In the future, this experimental approach will enable us to detect possible interacting effects. Additionally, we should not only focus on one generation in risk assessment studies but include at least the following generation as well. Further research is needed to understand how AgNPs affect the kairomone-mediated anti-predator defence mechanism in *Daphnia* species.

## Material and Methods

### Study species

For the experiments, we used the laboratory-cultured *Daphnia magna* (clone V) originally provided by the Federal Environment Agency (Berlin, Germany). *D. magna* were cultured in a temperature conditioned room (20 ± 1 °C) with a light:dark photoperiod of 16:8 h. ASTM reconstituted hard freshwater^36^, additionally enriched with selenium and vitamins (biotin, thiamine hydrochloride, cyanocobalamin)^37^ served as culture medium. Once a week the culture medium was renewed and juveniles were removed three times a week to avoid high density^12^. Test animals were fed daily with the green algae *Desmodesmus subspicatus* at a carbon concentration of 0.2 mg C/*D. magna*/day. Algae were cultured with an appropriate culture medium^38^ in an air conditioned room (24 ± 1 °C) under a 16:8 h light:dark photoperiod. Before use, algae were centrifuged, culture medium discarded and algae pellets resuspended with ultrapure water.

### Silver-nanoparticles (NM-300K)

In this study, we used NM-300K particles from the OECD Working Party on Manufactured Nanomaterials (WPMN) Sponsorship^39^ as AgNPs. The aqueous dispersion of NM-300K contained 10 w/w % silver and two stabilizing agents (4% each of Polyoxethylene Glycerol Trioleate and Polyoxyethylene (20) Sorbita mono-Laurat (Tween 20)) and had an average particle size of 15 nm^39^. The stability of NM-300K particles in ASTM medium (at equal concentrations as used in this study) shown by STEM analyses also performed at the University of Siegen (Germany) is documented by Hartmann *et al*.^12^ and Galhano *et al*.^40^. Based on these data, we can confirm that the reference material NM-300K is stable over 24 h (longest period without water exchange) and did not change in shape and size (analysed with the same material as used in this study). A S/TEM image of AgNPs (NM-300K) dispersed in ASTM medium, measured directly after the preparation of the stock solution is shown in Fig. S1.

To generate a homogenous suspension of AgNPs, the NM-300K stock vial was sonicated in an ultrasonic water bath for 10 minutes (Bransonic 221 ultrasonic cleaner, Branson Ultrasonic, USA) prior to use. A working stock with a nominal concentration of 50 mg/L was prepared in ASTM medium to set the test concentrations. As a matrix control, the AgNP-free stabilization agent NM-300K DIS was used. A dispersant stock solution was prepared accordingly. In this solvent with AgNP-free stabilization agent NM-300K DIS we diluted kairomones (see below) for Treatment I_b_.

### Preparation of kairomone stock medium

Kairomone stock medium (predator medium, PM) was prepared in accordance with Barbosa *et al*.^27^. In total, we used eight randomly selected adult wild-type zebrafish *Danio rerio* from West Aquarium GmbH (Bad Lauterberg, Germany) with a body length of about 40 mm and kept them in one 8 L glass tank filled with ASTM medium (without additional salts and vitamins) for 24 h in a temperature-controlled room (26 ± 1 °C) under a light-dark cycle of 14:10 hours. Fish were fed with 160 *D. magna* of varying sizes and ages one day before collecting the predator medium (PM). No extra fish flake food was given. After 24 h, when all *D. magna* were consumed by *D. rerio*, adult fish were returned to their home tank (80 ×40 ×35 cm³) and debris was allowed to settle down for 10 minutes before the medium, containing fish kairomones (predator medium) was directly used in the experiment. The predator medium (PM) was taken out from the glass tank with a 1 L glass beaker without any additional filtering. The freshly prepared PM was made every day under the same conditions as described above to ensure a high concentration of fish kairomones from *D. rerio* for the experiment. In their home tank, *D. rerio* was cultured in 112 L glass tanks (80 ×40 ×35 cm³) in groups of 100 animals with a sex ratio of 50:50 under a light-dark cycle of 14:10 hours and a water temperature of 26 ± 1 °C, a pH-value of 7–7.5 and a conductivity of 450 µS/cm. Water exchange (40%) took place two times a week. Water in the tank was aerated and filtered continuously. In their home tank, fish were fed daily in the morning with dry flake food (JBL GmbH & Co. KG, Germany), and additionally three times a week in the afternoon with brine shrimp *Artemia salina*.

### Experimental procedure and treatments

In this study, we followed the guidelines of the *D. magna* reproduction test^14^ and the method of Barbosa *et al*.^27^.^.^ In all experiments, a single *Daphnia* (maternal generation) was placed in a glass beaker (100 mL, Rotilabo, Carl Roth GmbH + Co. KG, Karlsruhe), filled with 50 ml of test medium. Each *D. magna* was less than 24 h old at the start of the experiment. In each treatment group, maternal *D. magna* (n = 12) were exposed for 21 days. The offspring were removed from the test vessel as soon as possible and kept in ASTM medium without AgNPs. Thus, offspring were not exposed to AgNPs and we did not perform a multi-generational study. Medium renewal took place daily to ensure a high kairomone concentration throughout the complete test period. The O_2_ (mg/L), pH and temperature (°C) of old and fresh medium for one test beaker of each treatment group were measured once a week with a WTW Multi 3430 (WTW GmbH, Weilheim, Germany). *Daphnia* were fed daily with green algae *Desmodesmus subspicatus* with 0.2 mg C/*D. magna*/day algae suspension. We determined ‘time to first brood’, ‘reproduction’ (as the number of offspring), ‘maternal body length (mBL)’ (as distance from naupliar eye to the base of the dorsal spine) and ‘maternal spine length (mSL)’, and calculated ‘relative spine length of maternal *Daphnia* (mRSL)’ after each moult and after 21 days at the end of the experiment. We checked the beaker for offspring daily. We removed offspring of each brood from the beaker as soon as possible and measured ‘offspring body length (oBL)’, ‘offspring spine length (oSL)’, and ‘relative spine length of offspring (oRSL)’ as morphological traits. We took pictures of body length and spine length with a digital camera (Nikon Coolpix L830, Chiyoda, Tokyo, Japan) and analysed pictures using the software AxioVision (Carl Zeiss, Jena). We performed the following controls and treatments:

I_a_. PM: Predator medium (PM) containing solely kairomones of *D. rerio* as a positive control for a kairomone induced response.

I_b_. PM + NM-300K DIS: Predator medium (PM) enriched with NM-300K DIS as a control to exclude possible effects of the stabilization agent.

II. PM + 2.5 µg Ag/L: Predator medium (PM) enriched with 2.5 µg/L of AgNPs.

III. PM + 5 µg Ag/L: Predator medium (PM) enriched with 5 µg/L of AgNPs.

IV. PM + 10 µg Ag/L: Predator medium (PM) enriched with 10 µg/L of AgNPs.

V. PM + 20 µg Ag/L: Predator medium (PM) enriched with 20 µg/L of AgNPs.

C. Control: ASTM culture media as a reference.

In a previous study^12^, we investigated effects of AgNPs alone without kairomones on reproduction in *D.magna* using the same AgNP material and same AgNP-concentrations as used in this study. We detected a clear negative effect of AgNPs on the reproductive success of adult *Daphnia* over six generations. Based on the results of our former study we did not test the effects of AgNPs alone without kairomones here again. Exposure to NM-300K DIS alone, however, did not affect any morphological or life history traits in *Daphnia*^12^. Thus, we did not perform this additional control here.

All experiments were performed in accordance with relevant German guidelines and regulations.

### Determination of total Ag in media samples

A single set (N = 1) of fresh and aged test media samples were collected during the 21-day test period to determine total Ag concentrations. The fresh media sample was taken on day 15 of the reproduction study and the aged media sample 24 h later (day 16), which represented the longest period without water exchange. The aqueous samples were stored at 4 °C prior to analysis. Total Ag content of the aqueous samples was determined with ICP-MS (inductively coupled plasma mass spectrometry; iCAP Qc, Thermo Fisher Scientific, Bremen, Germany). Prior to analysis, samples were taken out of the fridge and shaken for 30 minutes with a shaking machine (Edmund Bühler, Bodelshausen, Germany). The aqueous test samples were digested with concentrated nitric acid (70%, Analytical Reagent Grade, Fisher Scientific, Loughborough, UK) for 90 min and diluted 100 times to obtain a concentration of 2.9% (w/v) HNO_3_. Silver standard solution (Inorganic Ventures, Christiansburg, VA, USA) was used to calibrate the instrument on the same day, n = 10, ^107^Ag^+^ was measured, Indium (Inorganic Ventures, Christiansburg, VA, USA) served as an internal standard. All concentrations were calculated from calibration graphs using the internal standard correction. Limit of detection (LOD) and limit of quantification (LOQ) for ^107^Ag^+^ were 0.1 µg/L and 0.3 µg/L, respectively, depending on the experimental setup. The detailed experimental parameters are presented in Supplementary Table S1.

### Statistical analysis

The statistical analysis was performed using the statistical program R version 3.2.4^41^. For all parameters, we first compared parameters between maternal *Daphnia* from the control (ASTM medium, C) and from Treatment I_a_ (PM) to test whether *D. rerio* was a useful predator for testing anti-predator defence mechanism in maternal *D. magna*. Secondly, we analysed the differences between Treatment I_a_ (PM) and Treatments II – V (PM + different concentration of AgNPs), including Treatment I_b_ (PM + NM-300K DIS) to analyse the influence of PM in combination with AgNPs and to exclude possible effects of the dispersant agent on test animals (maternal *Daphnia*). For each treatment, we calculated the life-history parameters reproduction (cumulative mean number of offspring) ± standard deviation (sd), time to first brood (days ± sd), maternal body length (mBL; mm ± sd), offspring body length (oBL; mm ± sd), maternal spine length (mSL; mm ± sd), offspring spine length (oSL; mm ± sd), and checked the data for normal distribution (Shapiro-Wilk test) and for homogeneity of variances (Bartlett´s test). If both requirements met, we performed a one-way analysis of variances (ANOVA), followed by a Dunnett´s post hoc-test for multiple comparisons to test for statistical differences within treatments. Was one requirement not fulfilled, the nonparametric alternative, the Kruskal-Wallis test and afterwards the Dunn’s Test of multiple comparisons using rank sums^42^ was used. Because relative spine length of maternal *Daphnia* (mRSL) and relative spine length in offspring (oRSL) are bounded^27^, the data were analysed as dependent variables by using a ‘*glmer’* (Generalized Linear Mixed Effect Model) of the package *lme4*^43^. As fixed factor, we added treatment as the categorical variable to each model. Relative spine length of maternal *Daphnia* (mRSL) and relative spine length in offspring (oRSL) were modelled using a Gamma error distribution and a Log link function^27^. We included the number of moults and identity of test animals as nested random effects to the model. Model assumptions were checked visually. The p-values were adjusted with Bonferroni correction. Significant p-values were marked with asterisks (*P < 0.05, **P < 0.01, ***P < 0.001). All p-values are two tailed.

## Supplementary information

Supplementary Information.

## Data Availability

The data that support the findings of this study are available from the corresponding author upon reasonable request.

## References

[1] Fent, K. *Ökotoxikologie: Umweltchemie-Toxikologie-Ökologie*. (Georg Thieme Verlag, 2013).

[2] Geissen V (2015). Emerging pollutants in the environment: A challenge for water resource management. Int. Soil Water Conserv. Res..

[3] Benn TM, Westerhoff P (2008). Nanoparticle silver released into water from commercially available sock fabrics. Environ. Sci. Technol..

[4] Vogt R (2019). Spatiotemporal distribution of silver and silver-containing nanoparticles in a prealpine lake in relation to the discharge from a wastewater treatment plant. Sci. Total Environ..

[5] Gottschalk F, Nowack B (2011). The release of engineered nanomaterials to the environment. J. Environ. Monit..

[6] Maurer-Jones MA, Gunsolus IL, Murphy CJ, Haynes CL (2013). Toxicity of Engineered Nanoparticles in the Environment. Anal. Chem..

[7] Völker, C., Boedicker, C., Daubenthaler, J., Oetken, M. & Oehlmann, J. Comparative toxicity assessment of nanosilver on three *Daphnia* species in acute, chronic and multi-generation experiments. *Plos One* **8** (2013). 10.1371/journal.pone.0075026 PMC3792065 24116021

[8] Newton KM, Puppala HL, Kitchens CL, Colvin VL, Klaine SJ (2013). Silver nanoparticle toxicity to *Daphnia magna* is a function of dissolved silver concentration. Environ. Toxicol. Chem..

[9] Ribeiro F (2014). Silver nanoparticles and silver nitrate induce high toxicity to *Pseudokirchneriella subcapitata*, *Daphnia magna* and *Danio rerio*. Sci. Total Environ..

[10] Seitz F (2015). Effects of silver nanoparticle properties, media pH and dissolved organic matter on toxicity to *Daphnia magna*. Ecotoxicol. Environ. Saf..

[11] Bowman CR, Bailey FC, Elrod-Erickson M, Neigh AM, Otter RR (2012). Effects of silver nanoparticles on zebrafish (*Danio rerio*) and *Escherichia coli* (ATCC 25922): A comparison of toxicity based on total surface area versus mass concentration of particles in a model eukaryotic and prokaryotic system. Environ. Toxicol. Chem..

[12] Hartmann S (2019). Comparative multi-generation study on long-term effects of pristine and wastewater-borne silver and titanium dioxide nanoparticles on key lifecycle parameters in *Daphnia magna*. NanoImpact.

[13] OECD. *Test No. 202: Daphnia sp., Acute Immobilisation Test*. (2004).

[14] OECD. *Test No. 211: Daphnia magna Reproduction Test*. (OCED Publishing) (2012).

[15] Kim HJ, Koedrith P, Seo YR (2015). Ecotoxicogenomic Approaches for Understanding Molecular Mechanisms of Environmental Chemical Toxicity Using Aquatic Invertebrate, Daphnia Model Organism. *Int*. J. Mol. Sci..

[16] Wang P (2018). Metabolite changes behind faster growth and less reproduction of *Daphnia similis* exposed to low-dose silver nanoparticles. Ecotoxicol. Environ. Saf..

[17] Zhao C-M, Wang W-X (2011). Comparison of acute and chronic toxicity of silver nanoparticles and silver nitrate to *Daphnia magna*. Environ. Toxicol. Chem..

[18] Pokhrel LR, Dubey B (2012). Potential Impact of Low-Concentration Silver Nanoparticles on Predator–Prey Interactions between Predatory Dragonfly Nymphs and Daphnia magna as a Prey. Environ. Sci. Technol..

[19] Trotter B, Ramsperger AFRM, Raab P, Haberstroh J, Laforsch C (2019). Plastic waste interferes with chemical communication in aquatic ecosystems. Sci Rep.

[20] Laforsch C, Tollrian R (2004). Inducible Defense In Multipredator Environments: Cyclomophosis in *Daphnia cucullata*. Ecology.

[21] Rabus M, Laforsch C (2011). Growing large and bulky in the presence of the enemy: *Daphnia magna* gradually switches the mode of inducible morphological defences. Funct. Ecol..

[22] Laforsch C, Tollrian R (2004). Extreme helmet formation in *Daphnia cucullata* induced by small-scale turbulence. J. Plankton Res..

[23] Tollrian R (1993). Neckteeth formation in *Daphnia pulex* as an example of continuous phenotypic plasticity: morphological effects of Chaoborus kairomone concentration and their quantification. J. Plankton Res..

[24] Hunter K, Pyle G (2004). Morphological responses of *Daphnia pulex* to Chaoborus americanus kairomone in the presence and absence of metals. Environ. Toxicol. Chem..

[25] Petrusek A, Tollrian R, Schwenk K, Haas A, Laforsch C (2009). A “crown of thorns” is an inducible defense that protects *Daphnia* against an ancient predator. PNAS.

[26] Weiss, L., Laforsch, C. & Tollrian, R. The taste of predation and the defences of prey. *Chemical Ecology in Aquatic Systems*, 111–126 (2012).

[27] Barbosa M, Pestana J, Soares AM (2014). Predation life history responses to increased temperature variability. Plos One.

[28] Tollrian R (1995). *Chaoborus crystallinus* predation on *Daphnia pulex*: can induced morphological changes balance effects of body size on vulnerability?. Oecologia.

[29] Stibor, H. & Lüning, J. Predator-induced phenotypic variation in the pattern of growth and reproduction in *Daphnia hyalina* (Crustacea: Cladocera). *Funct. Ecol*., 97–101 (1994).

[30] Ślusarczyk M, Ochocka A, Biecek P (2013). Prevalence of kairomone-induced diapause in *Daphnia magna* from habitats with and without fish. Hydrobiologia.

[31] Hesse O, Engelbrecht W, Laforsch C, Wolinska J (2012). Fighting parasites and predators: How to deal with multiple threats?. BMC Ecol..

[32] Weber A, Declerck S (1997). Phenotypic plasticity of *Daphnia* life history traits in response to predator kairomones: genetic variability and evolutionary potential. Hydrobiologia.

[33] Weiss LC, Leimann J, Tollrian R (2015). Predator-induced defences in *Daphnia longicephala*: location of kairomone receptors and timeline of sensitive phases to trait formation. J. Exp. Biol..

[34] Zhang W (2019). Fate and toxicity of silver nanoparticles in freshwater from laboratory to realistic environments: a review. Environmental Science and Pollution Research.

[35] Hales NR (2017). Contrasting gene expression programs correspond with predator‐induced phenotypic plasticity within and across generations in. Daphnia. Mol. Ecol..

[36] ASTM. *In* ASTM International. West Conshohocken, PA, USA Vol. Standard E729 (2007).

[37] Seitz F, Bundschuh M, Rosenfeldt RR, Schulz R (2013). Nanoparticle toxicity in *Daphnia magna* reproduction studies: The importance of test design. Aquat. Toxicol..

[38] Bringmann G, Kühn R (1980). Comparison of the toxicity thresholds of water pollutants to bacteria, algae, and protozoa in the cell multiplication inhibition test. Water Res..

[39] Klein, C. *et al*. *NM-Series of representative manufactured nanomaterials: NM-300 Silver Characterisation, Stability*, *Homogeneity*. (2011).

[40] Galhano V (2020). Impact of wastewater-borne nanoparticles of silver and titanium dioxide on the swimming behaviour and biochemical markers of Daphnia magna: An integrated approach. Aquat. Toxicol..

[41] RCore Team. R: A language and environment for statistical computing. (2016).

[42] Dinno, A. Package ´dunn. test: Dunn’s test of multiple comparisons using rank sums´. *R foundation for statistical computing*, *Vienna* (2015).

[43] Bates, D. *et al*. Package ‘lme4’. R foundation for statistical computing, Vienna (2014).

