# Supplementary material for: Defective defence in Daphnia daughters: silver nanoparticles inhibit anti-predator defence in offspring but not in maternal Daphnia magna
Source: Sci Rep. 2020 May 15;10:8021. doi: 10.1038/s41598-020-64652-7 (PMC7229026; doi:10.1038/s41598-020-64652-7)
Supplement: Supplementary file 1 — Supplementary Information. [file 41598_2020_64652_MOESM1_ESM.pdf]

Defective defence in *Daphnia* daughters: silver nanoparticles inhibit anti-predator defence in offspring but not in maternal *Daphnia magna*

Sarah Hartmann[1], Anna Beasley[1,2], Darya Mozhayeva[3], Carsten Engelhard[3], Klaudia Witte*[,1]

[1]Research Group of Ecology and Behavioural Biology, Institute of Biology, Department of Chemistry and Biology, University of Siegen, Adolf-Reichwein-Strasse 2, Siegen, 57076, Germany.

[2]Faculty of Biology, Medicine and Health, University of Manchester, Oxford Road, Manchester, M13 9PL, United Kingdom.

[3]Department of Chemistry and Biology, University of Siegen, Adolf-Reichwein-Strasse 2, Siegen, 57076, Germany.

Corresponding author: witte@biologie.uni-siegen.de

Klaudia Witte: ORCID: 0000-0002-2812-9936

Sarah Hartmann: ORCID: 0000-0002-5714-342X

Darya Mozhayeva ORCID: 0000-0001-9831-7839

Carsten Engelhard ORCID: 0000-0002-7020-9278

Supplementary Information:

Table S1: ICP-MS instrumental parameters used for total Ag quantitation in aqueous test samples.

Parameter	Value
**ICP-MS**	iCAP Qc (Thermo Fisher Scientific, Bremen, Germany)
**Nebulizer**	C400d (Savillex, Eden Prairie, MN, USA)
**Spray chamber**	Peltier-cooled cyclonic quartz
**Radio-frequency power**	1550 W
**Ar cooling gas flow**	14 L/min
**Ar auxiliary gas flow**	0.8 L/min
**Ar nebulizer gas flow**	1.0 L/min
**Sampling position**	5 mm
**Skimmer type**	Ni (insert version)
**Torch injector inner diameter**	2.5 mm
**Dwell time**	10 ms
**Monitored isotope**	$^{107}Ag^{+}$

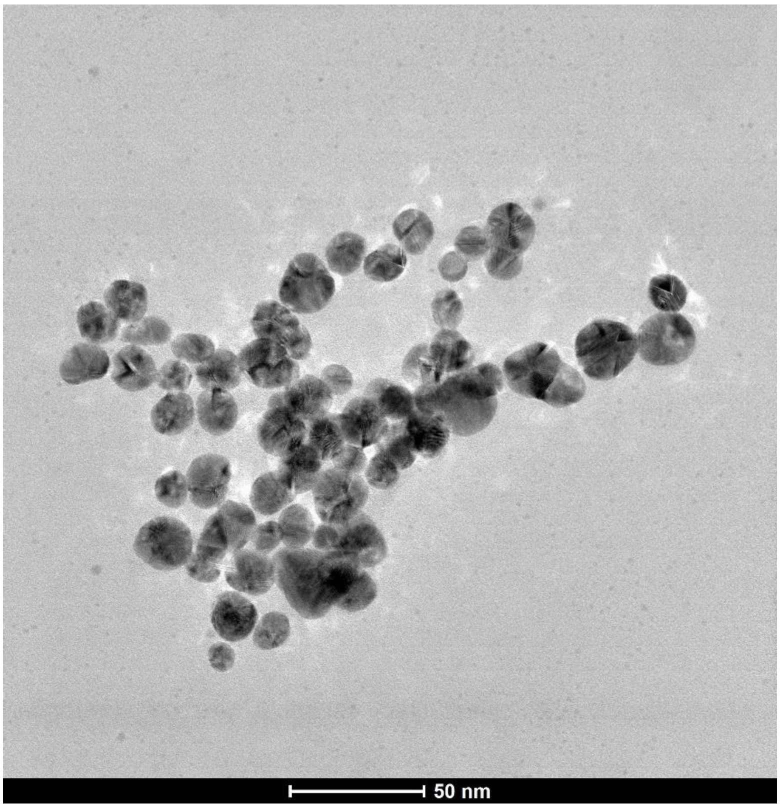

Figure S1: S/TEM image of AgNPs (NM-300K) dispersed in ASTM medium, measured directly after the preparation of the stock solution. A FEI Talos F200X electron microscope (Thermo Fisher Scientific, Waltham, USA) operating at 200 kV was used for (scanning) transmission electron microscopy (S/TEM) analysis. For more information see Hartmann et al.[12].

Table S2: Mean body length (mm ± sd) and mean spine length (mm ± sd) of adult *Daphnia magna* after each moult. Asterisks indicated significant differences compared to predator medium. * $P < 0.05$. $n = 12$.

Moult	mean body length (mm ± sd)						mean spine length (mm ± sd)					
	Treatment						Treatment					
	Predator medium (PM)	PM + 2.5 µg/L AgNPs	PM + 5 µg/L AgNPs	PM + 10 µg/L AgNPs	PM + 20 µg/L AgNPs	Control	Predator medium (PM)	PM + 2.5 µg/L AgNPs	PM + 5 µg/L AgNPs	PM + 10 µg/L AgNPs	PM + 20 µg/L AgNPs	Control
0	0.95 ± 0.09	1.04 ± 0.05	1.07 ± 0.11	0.98 ± 0.03	1.05 ± 0.13	0.97 ± 0.14	0.50 ± 0.02	0.53 ± 0.02	0.52 ± 0.04	0.46 ± 0.04	0.50 ± 0.04	0.50 ± 0.04
1	1.22 ± 0.19	1.35 ± 0.19	1.41 ± 0.22	1.23 ± 0.21	1.32 ± 0.35	1.33 ± 0.13	0.49 ± 0.08	0.52 ± 0.04	0.52 ± 0.04	0.47 ± 0.02	0.49 ± 0.09	0.44 ± 0.11
2	1.54 ± 0.31	1.69 ± 0.09	1.82 ± 0.27 *	1.48 ± 0.06	1.73 ± 0.22	1.68 ± 0.18	0.54 ± 0.09	0.55 ± 0.07	0.60 ± 0.06	0.51 ± 0.02	0.57 ± 0.03	0.47 ± 0.15
3	1.92 ± 0.40	2.15 ± 0.24	2.38 ± 0.54 *	1.80 ± 0.30	2.25 ± 0.32	2.15 ± 0.38	0.58 ± 0.10	0.63 ± 0.08	0.67 ± 0.10	0.55 ± 0.05	0.65 ± 0.03	0.52 ± 0.13
4	2.51 ± 0.46	2.73 ± 0.22	2.97 ± 0.43	2.26 ± 0.33	2.85 ± 0.26	2.67 ± 0.33	0.64 ± 0.16	0.71 ± 0.09	0.77 ± 0.10	0.63 ± 0.08	0.70 ± 0.06	0.57 ± 0.12
5	2.95 ± 0.43	3.21 ± 0.21	3.42 ± 0.39 *	2.76 ± 0.38	3.32 ± 0.20	3.17 ± 0.28	0.70 ± 0.15	0.79 ± 0.07	0.84 ± 0.08	0.72 ± 0.06	0.81 ± 0.06	0.64 ± 0.13
6	3.46 ± 0.32	3.63 ± 0.13	3.72 ± 0.31	3.26 ± 0.20	3.67 ± 0.14	3.44 ± 0.25	0.78 ± 0.14	0.84 ± 0.06	0.88 ± 0.08	0.78 ± 0.05	0.87 ± 0.04	0.68 ± 0.13
7	3.65 ± 0.23	3.81 ± 0.15	3.98 ± 0.30 *	3.63 ± 0.21	3.85 ± 0.23	3.62 ± 0.27	0.81 ± 0.15	0.85 ± 0.08	0.89 ± 0.09	0.83 ± 0.05	0.88 ± 0.06	0.70 ± 0.13
8	3.92 ± 0.23	4.09 ± 0.12	4.13 ± 0.20	3.83 ± 0.23	4.04 ± 0.24	3.71 ± 0.30	0.81 ± 0.16	0.88 ± 0.06	0.88 ± 0.11	0.78 ± 0.11	0.90 ± 0.09	0.69 ± 0.12

44
